# Using Machine Learning to Predict Complications in Pregnancy: A Systematic Review

**DOI:** 10.3389/fbioe.2021.780389

**Published:** 2022-01-19

**Authors:** Ayleen Bertini, Rodrigo Salas, Steren Chabert, Luis Sobrevia, Fabián Pardo

**Affiliations:** ^1^ Metabolic Diseases Research Laboratory (MDRL), Interdisciplinary Center for Research in Territorial Health of the Aconcagua Valley (CIISTe Aconcagua), Center for Biomedical Research (CIB), Universidad de Valparaíso, Valparaiso, Chile; ^2^ PhD Program Doctorado en Ciencias e Ingeniería para La Salud, Faculty of Medicine, Universidad de Valparaíso, Valparaiso, Chile; ^3^ School of Biomedical Engineering, Faculty of Engineering, Universidad de Valparaíso, Valparaiso, Chile; ^4^ Centro de Investigación y Desarrollo en INGeniería en Salud – CINGS, Universidad de Valparaíso, Valparaiso, Chile; ^5^ Instituto Milenio Intelligent Healthcare Engineering, Valparaíso, Chile; ^6^ Cellular and Molecular Physiology Laboratory (CMPL), Division of Obstetrics and Gynaecology, School of Medicine, Faculty of Medicine, Pontificia Universidad Católica de Chile, Santiago, Chile; ^7^ Department of Physiology, Faculty of Pharmacy, Universidad de Sevilla, Seville, Spain; ^8^ University of Queensland Centre for Clinical Research (UQCCR), Faculty of Medicine and Biomedical Sciences, University of Queensland, Herston, QLD, Australia; ^9^ Department of Pathology and Medical Biology, University of Groningen, University Medical Center Groningen, Groningen, Netherlands; ^10^ Medical School (Faculty of Medicine), São Paulo State University (UNESP), São Paulo, Brazil; ^11^ Tecnologico de Monterrey, Eutra, The Institute for Obesity Research, School of Medicine and Health Sciences, Monterrey, Mexico; ^12^ School of Medicine, Campus San Felipe, Faculty of Medicine, Universidad de Valparaíso, San Felipe, Chile

**Keywords:** perinatal complications, machine learning, pregnancy, artificial intelligence, predictive tool, prediction model

## Abstract

**Introduction:** Artificial intelligence is widely used in medical field, and machine learning has been increasingly used in health care, prediction, and diagnosis and as a method of determining priority. Machine learning methods have been features of several tools in the fields of obstetrics and childcare. This present review aims to summarize the machine learning techniques to predict perinatal complications.

**Objective:** To identify the applicability and performance of machine learning methods used to identify pregnancy complications.

**Methods:** A total of 98 articles were obtained with the keywords “machine learning,” “deep learning,” “artificial intelligence,” and accordingly as they related to perinatal complications (“complications in pregnancy,” “pregnancy complications”) from three scientific databases: PubMed, Scopus, and Web of Science. These were managed on the Mendeley platform and classified using the PRISMA method.

**Results:** A total of 31 articles were selected after elimination according to inclusion and exclusion criteria. The features used to predict perinatal complications were primarily electronic medical records (48%), medical images (29%), and biological markers (19%), while 4% were based on other types of features, such as sensors and fetal heart rate. The main perinatal complications considered in the application of machine learning thus far are pre-eclampsia and prematurity. In the 31 studies, a total of sixteen complications were predicted. The main precision metric used is the AUC. The machine learning methods with the best results were the prediction of prematurity from medical images using the support vector machine technique, with an accuracy of 95.7%, and the prediction of neonatal mortality with the XGBoost technique, with 99.7% accuracy.

**Conclusion:** It is important to continue promoting this area of research and promote solutions with multicenter clinical applicability through machine learning to reduce perinatal complications. This systematic review contributes significantly to the specialized literature on artificial intelligence and women’s health.

## Introduction

While most pregnancies and births are uneventful, all pregnancies are at risk. About 15% of all pregnant women will develop a life-threatening complication that requires specialized care, and some will require major obstetric intervention to survive (WHO, 2019). According to the World Health Organization (WHO), around 800 women die every day around the world from preventable causes related to the inherent risks of pregnancy. About 295,000 women died during and following pregnancy and childbirth in 2017. The vast majority of these deaths (94%) occurred in low-resource settings, and most could have been prevented (WHO, 2019).

Several maternal factors influence the appearance of perinatal complications. It is recognized that the first trimester of pregnancy is the best stage to predict and prevent perinatal complications. For example, it is known that increasing obesity in women of childbearing age leads to increased risk of perinatal complications such as gestational diabetes, large for gestational age (LGA), fetal macrosomia, and hypertensive syndromes in pregnancy (Denison et al., 2010; Mariona, 2016; Edwards and Wright, 2020). On the other hand, developed countries tend to see decreased birth rates over the years, leading to advanced gestational ages, predisposing women to adverse pregnancy outcomes (Laopaiboon et al., 2014).

Artificial intelligence (AI) technologies have been developed to analyze a wide range of health data, including patient data from multibiotic approaches, as well as clinical, behavioral, environmental, and drug data, and from various data included in the biomedical literature (Hinton, 2018). AI can help professionals in making decisions, reducing medical errors, improving accuracy in the interpretation of various diagnoses, and thereby reducing the workload to which they are exposed (Makary and Daniel, 2016). Machine learning (ML) is the subfield of computer science and a branch of AI. These techniques provide the ability to infer meaningful connections between data items from various data sets that would otherwise be difficult to correlate (Darcy et al., 2016; Obermeyer and Emanuel, 2016). Due to the large quantity and complex nature of medical information, ML is recognized as a promising method for supporting diagnosis or predicting clinical outcomes (Bottaci et al., 1997; Frizzell et al., 2017).

There are different types of data used for health learning models, including electronic medical records, medical images, biochemical parameters, and biological markers (Ahmed et al., 2020). The type of data that is used depends on what one tries to diagnose through ML.

Most of these decision support systems remain complex black boxes, which means that their internal logic is hidden from the clinical team who cannot fully understand the rationale behind their predictions. Interpretability is important before any health-care team can increase reliance on ML systems (Carvalho et al., 2019). Therefore, the research community has focused on developing both interpretable models and explanatory methods in recent years.

In general, the ML models are validated using the train–test split or the cross-validation schemes. Models are usually initially fitted to a training data set (Sohil et al., 2021), a set of examples used to fit the model parameters. Model fitting may include both variable selection and parameter estimation (Ripley, 1996). The test data set is a data set that is used to provide an unbiased evaluation of a final model fit on the training data set (Brownlee, 2017). Cross-validation is a statistical method for evaluating and comparing learning algorithms by dividing the data into k-folds, where each fold is separated into two segments: one used to learn or train a model and one used to validate the model. In typical cross-validation, the training and validation sets must be crossed in successive rounds so that each data point has a chance to be validated (Refaeilzadeh et al., 2009). Deciding the sizes and strategies for partitioning data sets into training, test, and validation sets depend mainly on the problem and available data. The performance metrics of the ML model are related to the ability of a test to determine if a health diagnosis is effective. Some of the commonly used metrics are accuracy (number of correctly classified assessments over the total number of assessments), precision, sensitivity and specificity, predictive values, probability ratios, and the area under the ROC curve (Šimundić, 2009). To evaluate the success of an ML system when predicting a medical diagnosis, these must be taken into account. It is relevant to note that the area under the curve (AUC) is one of the main performance metrics used in prediction systems; however, metrics such as precision are recommended to complement the results.

Recent studies have described how AI has been involved in areas like gynecology and obstetrics (Iftikhar et al., 2020; Cecula, 2021); however, the effect of all ML techniques on the prediction of perinatal complications has not been reviewed. Thus, we decided to carry out this review to present and synthesize different ML-based models, highlighting the main input characteristics used for training, output results, performance metrics in prediction, and contribution to decision-making related to perinatal complications associated with non-congenital risk factors in pregnant women.

## Methods

This systematic review was carried out following the guidelines for systematic reviews and meta-analysis (PRISMA) (Urrútia and Bonfill, 2010) (Supplementary Table S1).

### Information Sources and Search Strategy

Full and original articles related to ML techniques on complications during pregnancy published in English from 2015 to 2020 were searched on PubMed, Web of Science, and Scopus databases. Search terms were chosen and searches performed in an iterative process, initially using word headings associated with ML, such as “machine learning,” “deep learning,” “artificial intelligence,” and related to perinatal complications, such as “complications in pregnancy” and “pregnancy complications,” and excluding articles related to postpartum and congenital complications. For PubMed, the MESH terms were used to include associated synonyms in the search, and for Scopus and Web of Science, the terms of interest mentioned before with Boolean operators were used (Table 1). The search and final collection of articles were 98 articles, of which 20 were excluded by duplication.

**TABLE 1:** Search expressions used in the systematic review.

Data base	Search expression	Year of publication
PubMed	[“Machine learning” (Mesh)] AND “Pregnancy Complications” (Mesh) NOT (“postpartum”)	2015–2020
Web of Science	(“Machine learning” OR “Deep learning” AND (“complications in pregnancy” OR “pregnancy complications” OR “perinatal complications”) NOT (“postpartum”)	
Scopus		

### Eligibility Criteria

The included criteria for the articles searched were 1) English original articles, 2) access to full text, 3) studies based on humans, 4) studies using machine learning methods to predict complications in pregnancy, and 5) complications during pregnancy and at term in the mother and the newborn. The exclusion criteria applied were 1) systematic reviews, meta-analysis, and bibliographic reviews; 2) articles that included postpartum complications; 3) maternal congenital disease that increases the risk of perinatal complications; and 4) fetal congenital diseases. Articles were added manually according to the aforementioned criteria.

### Article Screening

All articles found were uploaded to the Mendeley desktop platform, where they were saved in a dedicated folder for the present systematic review. After eliminating the duplicate articles, a total of 78 articles remained. Then 16 articles were excluded by title, 18 were excluded by criteria, and 19 were excluded after reading. Finally, 31 articles for the review were selected. The selected articles were classified by the ML model used, type of features used, outputs, and performance metrics, in order to estimate which methods are the most accurate in the context of predicting perinatal complications.

### Risk of Bias

The 31 articles were subjected to the CASP checklist, which contains 11 questions to help evaluate a clinical prediction rule (CASP, 2017). Study quality was scored according to the CASP critical score: if the criterion was met entirely = 2 points; criterion partially met = 1 point; and criterion not applicable/not met/not mentioned = 0. Finally, study quality was ranked: a total score of 22 = high quality; 16–21 = moderate quality; and ≤15 = low quality.

### Data Synthesis and Visualization

To optimize the visualization of the results obtained in the systematic review, several tables were made according to the terms addressed in the search, showing complications that the models seek to predict, input characteristics for the training of the ML model, the type of ML used, and its validation and performance metrics.

## Results

### Study Characteristics

**FIGURE 1:**
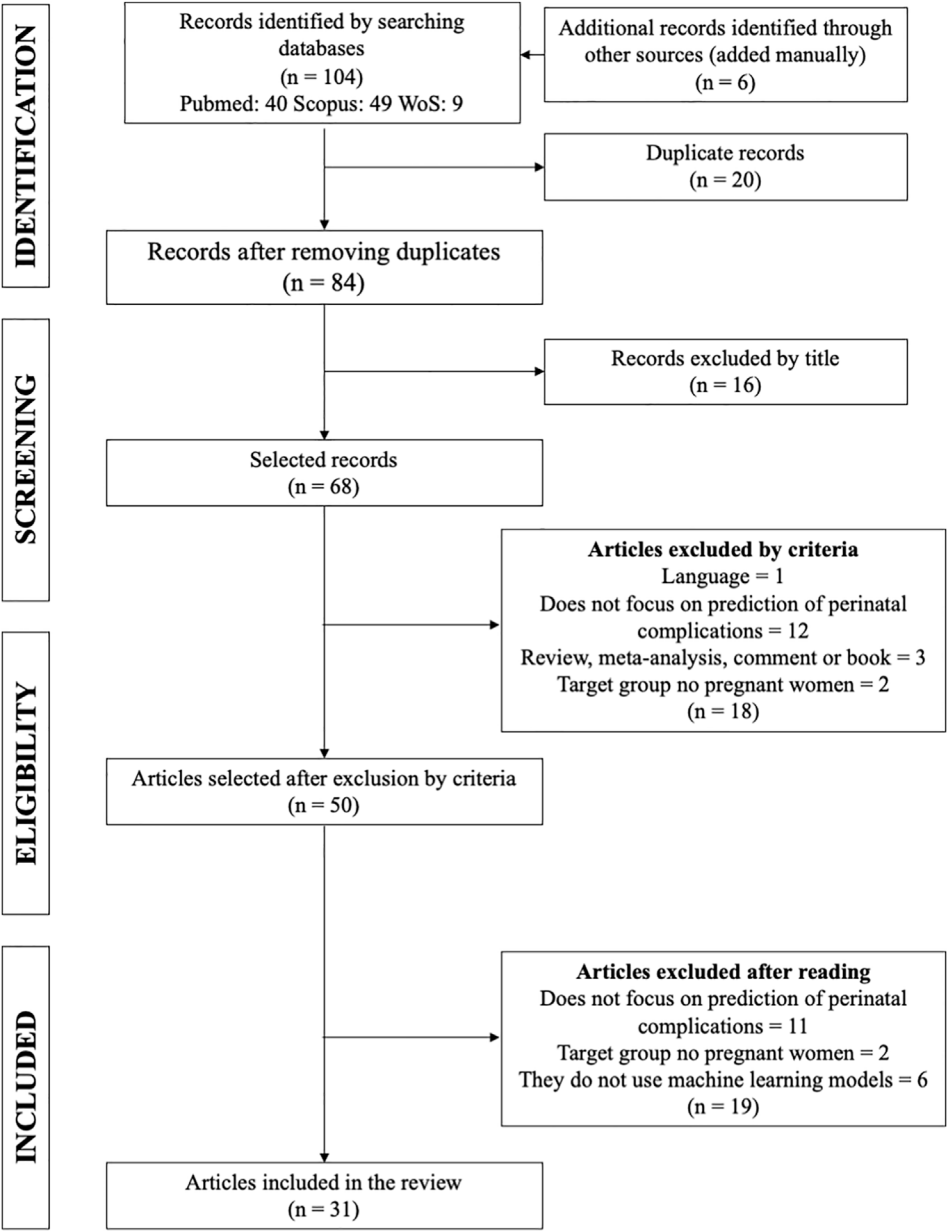
Process for selecting articles for the systematic review (PRISMA). One hundred four articles were found. Sixteen articles were excluded by title, 18 were excluded by criteria, and 19 were excluded after reading. Finally, 31 articles for the review were selected.

To apply the PRISMA method, the articles have been classified according to the criteria mentioned before: title, abstract, and the full article. A total of 84 articles were found, of which 52 were excluded because they did not meet the search criteria of interest. Of these, 16 were eliminated by title, 18 after reading the abstract, and 19 after reading the entire article, leaving 31 articles to analyze (Figure 1 and Supplementary Table S2). The type of studies in the manuscripts analyzed were mainly cohort (87.2%) and retrospective (96.8%). The populations studied were primarily from Asia and Europe (both 32.3%), followed by North and South America (22.5 and 6.5%, respectively). An increased rate of studies was observed during 2019 (35.5%) (Table 2). The features mainly used to predict perinatal complications are electronic medical records (48%) and then medical images (29%), biological markers (19%), and 4% are based on another type of feature, in this case, sensors (Moreira et al., 2016a) and fetal heart rate (Zhao et al., 2019). Two studies contemplate two features: electronic medical records and medical images (Nair, 2018; Lipschuetz et al., 2020).

**TABLE 2:** Main characteristics of selected articles.

Type of study	Temporality	Geographic location of the study group	Year of publication
Cohort (87.2%)	Retrospective (96.8%)	Asia (32.3%)	2015 (3.2%)
Control case (6.4%)	Prospective (3.2%)	Europe (32.3%)	2016 (9.6%)
Exploratory (3.2%)		North America (22.5%)	2017 (12.9%)
Cross section (3.2%)		South America (6.5%)	2018 (19.4%)
		Africa (3.2%)	2019 (35.5%)
		Oceania (3.2%)	2020 (19.4%)

According to the CASP checklist, one article met the total score and was classified as a high-quality article (Gao et al., 2019). The rest of the items were classified as moderate quality and none as low quality according to the evaluation criteria (average total score = 18–19). It is essential to mention that the “non-compliance” items were not being mentioned or not applicable to the study. The item asking whether the sample was randomized in 15 articles does not apply since analyzed retrospective electronic health records or images. Regarding using a comparison group, 12 reports do not apply due to retrospective data and data management for the prediction model (Supplementary Figure S1).

### Features Studied

The choice of informative, discriminatory, and independent characteristics is crucial to achieving effective algorithms for recognizing, classifying, and regression patterns. Thus, the four types of features analyzed in the articles were electronic medical records (EMRs) (Table 3), medical images (recordings, ecotomographs, ultrasound, resonance, etc.) (Table 4), biological markers (Table 5), and others (sensors and fetal heart rate) (Table 6).

**TABLE 3:** Perinatal complications predicted through ML models using electronic medical records.

Electronic medical records									
Ref	Time of data collection	Number of records	Outcome	Validation technique	ML methods	Performance metrics			
						AUC	Sen. (%)	Spec. (%)	Acc. (%)
Lipschuetz et al. (2020)	During pregnancy with term delivery	9,888	TOLAC failure risk	10-fold cross-validation and deletion of a portion of the data	Gradient increasing machines	0.793	—	—	—
			-High		RF	0.756	—	—	—
			-Medium		RF	0.782	—	—	—
			-Low		AdaBoost set	0.784	—	—	—
Hamilton et al. (2020)	<22 gw	100	Severe neonatal mortality v/s no severe neonatal mortality	10 replicates of 10-fold cross-validation and on the one standard error rule	Decision tree	0.853	79.7	80.9	75.6
					SVM	0.851	79.1	79.6	77.4
					Generalized additive model	0.850	80.6	81.8	75.0
					Simple neural network	0.848	78.5	80.7	73.3
Artzi et al. (2020)	<20 gw	588,622	High-risk GDM v/s low-risk GDM	Cross-validation on the training set, and resampling from the validation	Gradient augmentation machine built with decision tree base learners	0.850	—	—	—
Jhee et al. (2019)	Early second trimester to 34 gw	1,006	Pre-eclampsia v/s no pre-eclampsia	Training (70%) validation set (30%)	Logistic regression	—	70.3	—	86.2
					Decision tree	—	64.8	—	87.4
					Naive Bayes	—	50	—	89.9
					SVM	—	13.7	—	89.2
					RF	—	67.9	—	92.3
					Stochastic gradient augmentation method	—	60.3	—	97.3
Rittenhouse et al. (2019)	During pregnancy (not specified)	1,450	Premature v/s not premature^a^	k-fold cross-validation (with 10 folds)	Binary logistic regression model, RF classification, and generalized additive model	0.868	98.9	—	—
		—	Gestational age prediction	k-fold cross-validation (with 10 folds)	Combined continuous model of linear regression, RF, regression, and generalized additive models	0.878	90.2	—	—
Kuhle et al. (2018)	Pre-pregnancy at 26 gw	30,705	LGA v/s AGA	Test (20%) training (80%) and ten-fold cross-validation in the training data	RF	0.728	—	—	79.9
					Decision tree	0.718	—	—	79.4
					Elastic net	0.748	—	—	80.9
					Gradient increasing machines	0.748	—	—	80.5
					Logistic regression	0.745	—	—	81.3
					Neural network	0.746	—	—	81.2
			SGA v/s AGA	Test (20%) training (80%) and ten-fold cross-validation in the training data	RF	0.745	—	—	90.3
					Decision tree	0.713	—	—	80.1
					Elastic net	0.771	—	—	91.2
					Gradient increasing machines	0.766	—	—	91.1
					Logistic regression	0.771	—	—	91.2
					RF	0.772	—	—	91.4
Khatibi et al. (2019)	During pregnancy, before 37 gw	1,547,677	Non-premature delivery v/s premature	Training dataset	Set of decision trees, SVM and RF	0.68	—	—	81.0
Malacova et al. (2020)	During pregnancy (not specified)	952,813	Miscarriage v/s born alive	Dataset was randomly divided into 10 folds	Artificial neural networks: multilayer perceptron + radial base networks	—	80	94.1	90.9
Pan et al. (2017)	During pregnancy (not specified)	6,457	Adverse delivery v/s non-adverse delivery	10-fold cross-validation and repeated the cross-validation process with new folds 9 more times in the test set	Logistic regression	—	31.9	—	—
					Linear discriminant analysis	—	31.7	—	—
					RF	—	30.1	—	—
					Naive Bayes	—	29.2	—	—
Moreira et al., 2016b	During pregnancy (not specified)	25	Hypertensive disorder v/s without hypertensive disorder	10-fold cross-validation for decision trees	Decision tree J48	0.748	60	—	—
				5-fold cross-validation method	Naive Bayes	0.782	52	—	—
Gao et al. (2019)	During pregnancy (not specified)	45,858	Severe maternal morbidity v/s no serious maternal morbidity	Train dataset and 10-fold stratified cross-validation	Logistic regression	0.937	76.5	—	—
Mailath-Pokorny et al. (2015)	Between 22 and 32 gw	617	Delivery prediction within 48 h of transfer v/s Before 32 gw	Validation set	Multivariate logistic regression	0.850	—	—	—
Shigemi et al. (2019)	Data from the first and last prenatal checkup	15,263	Macrosomia v/s No macrosomia	Training dataset (90%) and a validation dataset (10%)	Logistic regression	0.880	88	55	—
					RF	0.990	60	82	—
Paydar et al. (2017)	Before the first trimester	149	Live births v/s stillbirths	Test (70%) training (30%)	Logistic regression	0.834	40.5	99.7	94.7
					Decision tree	0.808	40.6	94.7	99.7
					RF	0.836	41.1	94.7	99.7
					XGBoost	0.842	45.3	94.7	99.7
					Artificial neural networks multilayer perceptron	0.840	43.5	94.7	99.7
			Spontaneous preterm birth		Multivariate logistic regression	0.670	—	—	—
Boland et al. (2017)	Each trimester of pregnancy	36,898	Pregnancies without congenital abnormality v/s pregnancies with congenital abnormality	Method of data validation is not identified	RF	—	—	—	88.9

Ref., references; ML, machine learning; AUC, area under curve; Sen, sensitivity; Spec, specificity; Acc, accuracy; TOLAC, trial of labor after caesarean, RF, random forest; gw, gestational weeks; SVM, support vector machine; GDM: gestational diabetes mellitus; LGA, large for gestational age; AGA, adequate for gestational age, SGA, mall for gestational age.

a This study also uses biological markers.

**TABLE 4:** Perinatal complications predicted through ML models using medical images.

Medical Images									
Ref	Time of data collection	Number of records	Outcome	Validation technique	ML methods	Performance metrics			
						AUC	Sen. (%)	Spec. (%)	Acc. (%)
Sun et al. (2019)	After 24 gw	155	Placental invasion v/s placenta previa simple	Test (83%) Training (17%)	Genetic algorithm-based machine learning algorithm implemented in TPOT	0.980	100	88.5	95.2
Chen et al. (2019)	150 EHG in pregnancy (not specified) and 150 EHG in labor (24 h before delivery usually)	300	Premature v/s born of term	Test (67%) training (33%)	Stacked sparse autocoder	0.900	92	88	90
					Extreme learning machine	0.840	80	88	83
					SVM	0.850	88	82	85
Fergus et al. (2018)	>36 gw	552	Vaginal delivery v/s caesarean section	Test (80%) training (30%)	SVM RF and linear discriminant analysis of features	0.960	87	90	
						—	—	—	—
Borowska et al. (2018)	From 24 to 28 gw	20	Deliver after 7 days v/s deliver within 7 days	10-fold cross-validation	PCA + SVM	—	—	—	83.32
					RQA + SVM	—	—	—	79.3
Veeramani and Muthusamy (2016)	During pregnancy (not specified)	ni	Diagnosis of recurrent lung diseases in the newborn	Test Training	RVM	—	—	—	100
					Multilevel RVM	—	—	—	90
Romeo et al. (2019)	During pregnancy (not specified)	108	Delivery with placental accreta spectrum v/s delivery without placental accreta spectrum	Test (75%) training (25%) and a 10-fold cross-validation	RF	—	93.7	93.7	95.6
					K-nearest neighbor	—	97.5	98.7	98.1
					Naive Bayes	—	86.1	75	80.5
		—			Multilayer perceptron	—	92.4	83.8	88.6
Sadi-Ahmed et al. (2017)	Between the 27th and the 32nd gw	30	Premature vs. term	100 iterations of “holdout” cross-validation for training and test sets	SVM	0.952	98.4	93	95.7
Cömert et al. (2018)	During pregnancy (not specified)	552	Presence of fetal hypoxia v/s absence of fetal hypoxia	Test (90%) training (10%) and 10-fold cross-validation	Least squares support vector machines	—	63.5	65.9	65.4
Weber et al. (2018)	First prenatal visit	∼2,700,000	Born preterm v/s born of term in white women v/s color	Test set and 5-fold cross-validation	Logistic Regression	0.625	56	62.5	—

Ref., references; ML, machine learning; AUC, area under curve; Sen, sensitivity; Spec, specificity; Acc, accuracy; gw, gestational weeks; TPOT, tree-based pipeline optimization tool; EHG, electrohysterograhic; SVM, support vector machine; PCA, principal components analysis; RQA, recurrence quantification analysis; RVM, relevance vector machine.

**TABLE 5:** Perinatal complications predicted through ML models using biological markers.

Biological Markers									
Ref	Time of data collection	Numbers of records	Outcome	Validation technique	ML methods	Performance metrics			
						AUC	Sen. (%)	Spec. (%)	Acc. (%)
Guo et al. (2020)*	For GDM <18 gw	2,199	GDM	Training and validation	Logistic regression	0.732	—	—	72.6
	For PE		PE			0.813	—	—	81.5
	<20gw		MA			0.766	71	82.3	80.0
	For MA and FGR, 12–28 gw		FGR			0.775	—	—	79.5
Liu et al. (2019)	>20 gw	77	PE v/s control	Test and training	SVM	0.958	95	66.7	—
Nair (2018)	>20 gw	38	PE v/s control	Test (85%) training (15%)	Artificial neural networks multilayer perception	0.908	—	—	—
Yoffe et al. (2019)	First trimester of gestation	43	GDM v/s without GDM^a^	Trained and evaluated the datasets via a leave-one-out cross-validation	Logistic regression	0.740	88	40	76
					RF	0.810	94	40	81
					AdaBoost	0.770	94	60	86
Munchel et al. (2020)	Between 12 and 37 gw	113	Severe PE v/s without PE	Dataset trained with 10-fold stratified cross-validation	AdaBoost	0.964	88	92	89

Ref., references; ML, machine learning; AUC, area under curve; Sen, sensitivity; Spec, specificity; Acc, accuracy; GDM, gestational diabetes mellitus; gw, gestational weeks; PE, pre-eclampsia; MA, macrosomia; FGR, fetal growth restriction; SVM, support vector machine.

a This study also uses electronic medical records.

**TABLE 6:** Perinatal complications predicted through ML models using sensors and fetal heart rate.

Other features									
Ref.	Time of data collection	Numbers of records	Outcome	Validation technique	ML methods	Performance metrics			
						AUC	Sen. (%)	Spec. (%)	Acc. (%)
Moreira et al., 2016a	During pregnancy (not specified)	25	Complication in hypertensive disorder v/s without complication in hypertensive disorder^a^	Leave-one-out method of cross-validation	Naive Bayes	0.687	42.3	94.4	80
Zhao et al. (2019)	Intrapartum	552	Presence v/s absence of fetal acidemia^b^	Training set and 10-fold cross-validation	Deep convolutional neural network	0.978	98.2	94.9	98.4

Ref., references; ML, machine learning; AUC, area under curve; Sen, sensitivity; Spec, specificity; Acc, accuracy.

a Sensors.

b Fetal heart rate.

### Perinatal Complications to Predict

**FIGURE 2:**
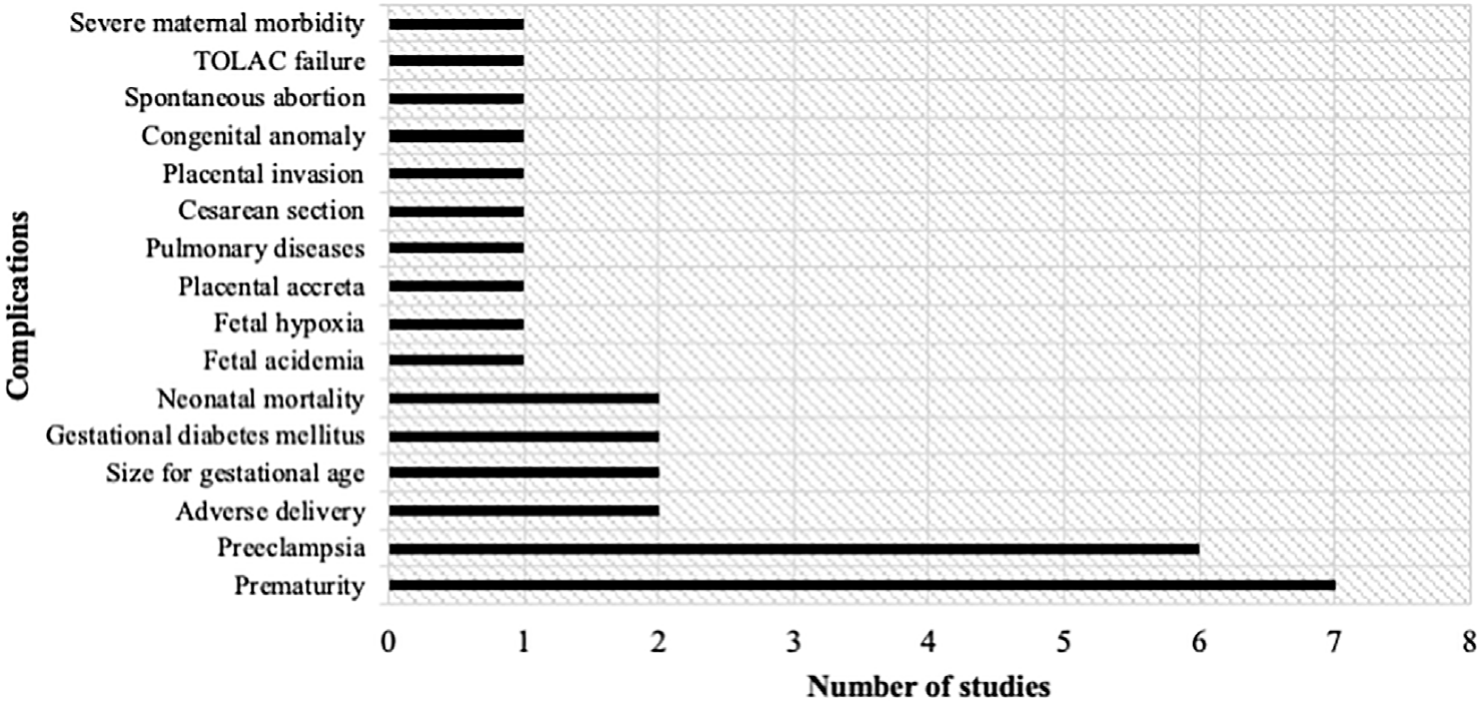
Number of studies according to the complication to be predicted. Sixteen complications were identified: Prematurity, pre-eclampsia, adverse delivery, size for gestational age, gestational diabetes mellitus, neonatal mortality, fetal acidemia, fetal hypoxia, placental accreta, pulmonary diseases, cesarean section, placental invasion, congenital anomaly, spontaneous abortion and trial of labor after cesarean (TOLAC) failure, and severe maternal morbidity.

These have been divided into 16 main prediction outputs: prematurity, pre-eclampsia, adverse delivery, size for gestational age, gestational diabetes mellitus, neonatal mortality, fetal acidemia, fetal hypoxia, placental accreta, pulmonary diseases, cesarean section, placental invasion, congenital anomaly, severe maternal morbidity, spontaneous abortion, and trial of labor after cesarean (TOLAC) failure (Figure 2). The main perinatal complications considered in the application of ML are prematurity (7 studies) and pre-eclampsia (6 studies).

### Validation Methods

Validation methods are strategies that allow the estimation of the predictive capacity of ML models. Fifty-five percent use training tests and the cross-validation method as a validation method with greater reliability in results, while 41.8% use a single validation method and 3.2% do not use any validation method (neither training tests nor cross-validation).

### ML Models and Performance Metrics

In the present review, 67.7% of the articles used AUC and 61.3% used the accuracy metric. Sensitivity was only evaluated in 61.3% of the studies. While all studies assess results with at least one performance metric, reports of predictive accuracy were often incomplete, with a total of 38.7% of studies reviewing at most two performance methods. According to the studies, none had a clinical application, they only functioned to establish precise prediction systems in the diagnosis of the different perinatal complications presented.

Twenty-one different ML methods were used to predict these 16 perinatal complications. Placental invasion is referred to as placental adhesive disorders observed in women with placenta previa or prior cesarean section that lead to complications such as perinatal hemorrhage and visceral injuries, where an early diagnosis is necessary for appropriate treatment (Sun et al., 2019). Excellent performance of placental invasion can be observed with an AUC and an accuracy of 0.980 and 95.2%, respectively, using the Tree-based Pipeline Optimization Tool (TPOT) (Sun et al., 2019). To predict fetal acidemia, using convolutional neural networks, an AUC and accuracy of 0.978 and 98.4% are achieved, respectively (Zhao et al., 2019). Only one study of the six attempting to diagnose pre-eclampsia had a performance considered as good, using the AdaBoost model, with an AUC of 0.964 and an accuracy of 89% (Munchel et al., 2020). The prediction of prematurity has excellent results in two studies; the one that uses SVM achieves an AUC of 0.952 and an accuracy of 95.7% (Sadi–Ahmed et al., 2017), and the study that uses stacked sparse autoencoder achieves an AUC of 0.900 and an accuracy of 90% (Chen et al., 2019). For the prediction of neonatal mortality, through sociodemographic records using XGBoost, an AUC of 0.842 and an accuracy of 99.7% were obtained (Hamilton et al., 2020). Regarding the performance of the predictions included in the greatest number of studies, prematurity outperformed pre-eclampsia according to the AUC (Table 7).

**TABLE 7:** Models with best performance according to AUC and accuracy.

Prediction	Input characteristics	ML model	Performance	No of pregnant women
Placental invasion	Magnetic resonance	TPOT	AUC: 0.980 – Acc: 95.2%	100–1,000
Fetal academia	Maternal sociodemographic characteristics	Neural networks	AUC: 0.978 – Acc: 98.4%	100–1,000
Pre-eclampsia	Biological marker	AdaBoost	AUC: 0.964 – Acc: 89%	<100
Prematurity	EHG recordings	SVM	AUC: 0.952 – Acc. 95.7%	100–1,000
Prematurity	EHG recordings	Stacked sparse autocoder	AUC 0.900 – Acc: 90%	100–1,000
Neonatal mortality	Maternal sociodemographic characteristics	XGBoost	AUC: 0.842 – Acc: 99.7%	>10,000

ML, machine learning; TPOT, tree-based pipeline optimization tool; AUC, area under curve; Acc, accuracy; EHG, electrohysterogram; SVM, support vector machine.

It was decided to corroborate the performance of the methods based on deep learning. Only four studies used deep learning methods. They all had an excellent performance. For the prediction of fetal acidemia, a deep convolutional network was used with an AUC of 0.978 and an accuracy of 98.4% (Zhao et al., 2019). For the prediction of spontaneous abortion, multilayer perceptron and radial-based networks were used, with an accuracy of 90.9% (Paydar et al., 2017). And as mentioned above, for the prediction of pre-eclampsia, using biological markers and multilayer perceptron, an AUC of 0.908 was obtained (Nair, 2018). For the prediction of neonatal mortality, through sociodemographic records using XGBoost, an AUC of 0.842 and an accuracy of 99.7% were obtained (Hamilton et al., 2020) (Table 8).

**TABLE 8:** Models and precision based on deep learning.

Prediction	Input characteristics	Deep learning model	Performance	N° of pregnant women
Fetal acidemia	Maternal and newborn sociodemographic characteristics	Deep convolutional network	AUC: 0.978, Acc: 98.4%	100 - 1,000
Spontaneous abortion	Maternal sociodemographic characteristics	Multilayer Perceptron and radial-based networks	Acc: 90.9%	100 - 1,000
Pre-eclampsia	Biological markers	Multilayer Perceptron	AUC: 0.908	<100
Neonatal mortality	Maternal sociodemographic characteristics	Multilayer Perceptron	AUC: 0.84 - Acc: 99.7%	>100,000

AUC, area under curve; Acc, accuracy.

### Interpretable ML Models

The interpretability of ML models refers to the degree to which a human being can consistently predict the outcome of the model (Kim et al., 2016), which has been well accepted by the clinical team. In this systematic review, we found that 24% of the studies use AI-interpretable ML models. The ML methods that were the most used in the prediction of perinatal complications were the random forest, logistic regression, neural networks, and support vector machine (SVM).

### Predictive Variables

Forty-eight percent of the studies explain the main characteristics of pregnant women that could be relevant to predict some conditions. Characteristics and antecedents such as gestational diabetes, cardiovascular disease, underlying diseases, and the age of the mother, as well as the presence of chronic arterial hypertension, are considered high-ranking features for the prediction of premature births; and the father’s nationality is very important to differentiate the provider-initiated spontaneous preterm births (Khatibi et al., 2019).

On the other hand, important predictors to determine the likelihood of a newborn to be small for gestational age (SGA) were smoking, a particular amount of gestational weight gain, and low–birth weight newborn. The body mass index (BMI) before pregnancy, gestational weight gain, and a macrosomic newborn in a previous delivery were the strongest predictors to determine large for gestational age (LGA) newborns (Kuhle et al., 2018). To predict fetal macrosomia, the determining variables were age ≥30, multiparity, 12 kg of total weight gain during pregnancy, abdominal circumference >95 cm (at the last perinatal checkup), and a gestation period over 39 weeks (Shigemi et al., 2019).

In order to predict pre-eclampsia, the most influential variables were systolic blood pressure, serum levels of ureic nitrogen and creatinine, platelet count, serum potassium level, leukocyte count, blood glucose level, serum calcium, and proteinuria levels in the early second trimester (Jhee et al., 2019). Interestingly, high pre-pregnancy BMI and previous preterm births (Pan et al., 2017) were able to predict whether pregnant women will have an adverse pregnancy outcome (preterm, low birth weight, neonatal/infant death, stay in the neonatal intensive care unit) and indicate the main risk characteristics.

Furthermore, in order to predict TOLAC, the determining factors in the prediction model were parity, age, vaginal birth with cesarean section in the past, gestational weeks, minimum gestation week in previous deliveries, the weight of the newborn from the previous delivery, dilation, and head position (Lipschuetz et al., 2020). To predict pregnancy complications associated with placental alterations (pre-eclampsia, GDM, fetal growth restriction, macrosomia), maternal age, BMI, newborn weight, and the results of adverse events in previous pregnancies were the most influential characteristics in the study (Guo et al., 2020).

To predict gestational age at delivery (if the newborn will be preterm) variables such as the date of the mother’s last menstruation, birth weight, delivery of twins, maternal height, hypertension during labor and HIV serological status were decisive in the ML model (Rittenhouse et al., 2019). To determine preterm birth, the presence of premature rupture of membranes and/or vaginal bleeding, ultrasound cervical length, gestation week, fetal fibronectin, and serum C-reactive protein were the determining variables (Mailath-Pokorny et al., 2015). In another study, prediction of preterm birth considered the most relevant variables to be maternal age, whether the mother was black, Hispanic, Asian, born in the United States, delivered by herself or assisted by a physician, presence of diabetes mellitus, chronic arterial hypertension, thyroid dysfunction, asthma, previous stillbirth, fetal weight loss, *in vitro* fertilization, nulliparity, being a smoker during the first trimester, and BMI (Weber et al., 2018).

Stillbirth can potentially be identified prenatally considering the combination of current pregnancy complications, congenital anomalies, maternal characteristics, and medical history (Malacova et al., 2020). Determining factors for the prediction of fetal acidemia were maternal age, gestational age, pH, extracellular fluid deficit, pCO2, base excess, APGAR 1 and 5 min, parity, gestational diabetes, birth weight, child sex, and the type of delivery (Zhao et al., 2019).

In the case of the prediction of severe maternal morbidity, the following characteristics were determining factors: ventilator dependence, intubation, critical care, acute respiratory failure, ventilation, trauma and postoperative pulmonary failure, fluid and electrolyte disorder, systemic inflammatory response syndrome, acidosis, and septicemia (Gao et al., 2019).

### Clinical Applicability of ML Systems

According to the studies, none had clinical application; they only served to establish precise prediction systems to diagnose the perinatal complications presented.

## Discussion

### Input Variables on Machine Learning

Machine learning plays a vital role and offers solutions with many applications, for example, image detection, data mining, natural language processing, and disease diagnosis (Maity and Das, 2017). This systematic review provides a study of different ML techniques for the diagnosis of different perinatal complications and frames a contribution to women’s health. A total of sixteen perinatal complications predicted by various ML models were detected, among which the most studied were prematurity and pre-eclampsia.

ML can significantly improve health care; however, it is necessary to consider the disadvantages of AI in health. Ethical dilemmas need to be addressed and the potential for human biases when creating computer algorithms (Ho et al., 2019). Health-care predictions can vary based on race, genetics, gender, and other characteristics, which could lead to the overestimation or underestimation of patient risk factors if not considered. When it comes to AI analysis in health care, it will be the physician’s responsibility to ensure that AI algorithms are developed and applied appropriately (Jordan and Mitchell, 2015).

In the present systematic review, the main data collection method was the use of electronic medical records. ML techniques can establish patterns from a data set based on electronic medical records (EMRs). Pattern recognition from these records supports in predicting and making decisions for diagnosis and treatment planning (Johnson et al., 2016). The application of EMR-based ML methods can be combined with other sources of large medical data, such as genomics, and medical imaging, which through predictive algorithms could improve clinical diagnosis and treatment systems, when used as complementary information (Barak-Corren et al., 2017). EMR data usually include demographics data, diagnoses, biochemical markers, vital signs, clinical notes, prescriptions, and procedures, which are generally easy to obtain and reduce transfer errors when handling large amounts of information. Previously, several studies have described medical diagnosis prediction tools mediated EMRs (McCoy et al., 2015; Osborn et al., 2015; Nguyen et al., 2017; Rajkomar et al., 2018); furthermore, in the present systematic review, 48% of the features for the diagnosis prediction model to perinatal complications came from EMRs, of which the most used features were sociodemographic maternal characteristics. Thus, this tool can predict perinatal complications common in a given population, contributing to the overall improvement of perinatal public health.

### Perinatal complications as Output Variables

Output variables were usually binary outputs (with complication or without complication). However, some studies quantified the risk, for example, the risk of TOLAC was classified as high, medium, or low (Lipschuetz et al., 2020), and in studies of gestational diabetes, one article quantified it as high risk or low risk (Cömert et al., 2018). The most frequently predicted perinatal complications in ML models were prematurity and pre-eclampsia. According to the literature, the high rate of preterm birth is a public health problem, since these newborns suffer substantial morbidity and mortality in the neonatal period, which translates to high medical costs (McCormick et al., 2011). Pre-eclampsia is a pregnancy disorder characterized by the new onset of hypertension after 20 weeks gestation and organ damage with underlying causes being endothelial dysfunction (ACOG (American College of Obstetricians and Gynecologists), 2020; Carrasco-Wong et al., 2021; Roberts, 1998). It is the leading cause of maternal and neonatal mortality and morbidity (Salsoso et al., 2017; Fondjo et al., 2019). Thus, prediction of the risk for developing pre-eclampsia can be performed in the first half of pregnancy.

### Performance of the Machine Learning Methods

Diagnostic accuracy is the ability of a test to discriminate between the target condition and health. This discriminative potential can be quantified by several performance tools, such as sensitivity and specificity, AUC, accuracy metric, and other measurements (Šimundić, 2009). While all studies assess results with at least one performance metric and just 38.7% assess at least two performance methods, reports of predictive accuracy were often incomplete. With this observation, it is imperative to show the same performance tools on the different prediction models to evaluate accuracy compared between them.

In this systematic review, several ML methods were used. One of the better performances was obtained by the Tree-based Pipeline Optimization Tool (TPOT) to predict placental invasion (Sun et al., 2019), which was previously used in the investigation of novel characteristics in data science, providing optimization of the studied parameters (Le et al., 2020). Another excellent performance observed was the convolutional neural network (CNN) to predict fetal acidemia (Zhao et al., 2019). The CNN has gained much attention from attempts made at harnessing its power to automatically learn intrinsic patterns from data, which can avoid time-consuming manual functions engineering, and capture hidden intrinsic patterns more effectively (Oquab et al., 2014). Moreover, in the health-care field, CNN has been shown to capture more hidden data patterns and learn high-level abstraction in problem-solving (Zhang et al., 2017).

It is essential to mention that it is difficult to reach a consensus on the best method for predicting perinatal complications, since not all of them had the same input variables, type of records, and a number of samples. However, the best performance metrics observed were the prediction model of prematurity from medical images using the SVM technique with an accuracy of 95.7% and the prediction of neonatal mortality using the XGBoost technique with an accuracy of 99.7%. SVM has shown simplicity and flexibility to address several classification problems and also offers balanced predictive performance even in studies where sample sizes may be limited (Alkhaleefah and Wu, 2018). The XGBoost technique is a very effective and widely used ML method that data scientists use to achieve state-of-the-art results in many ML challenges (Wang et al., 2020).

### Interpretability of Machine Learning

Despite the recognition of the value of ML in medical care, impediments persist for its greater acceptance within medical teams (Holzinger et al., 2019). A fundamental impediment relates to the nature of the black box, or “opacity,” of many ML algorithms. The term refers to a system in which only the inputs and outputs are observable, while the question of what is transforming the inputs into the outputs cannot be fully understood (Molnar, 2019). Therefore, new techniques have been developed to facilitate the understanding of the internal functioning of the model, granting interpretability, which seeks to provide transparency to the black box (Freitas, 2014; Doshi-Velez et al., 2017; Lipton, 2018), so that the end-user can understand the model and may even improve the ML system (Freitas, 2014). The improvement in the precision of the prediction will depend on the interpretability of the model to be used. This means that with ML interpretability, clinical staff could know which variables are involved in the prediction of a diagnosis.

Regarding the predictive variables, while most of them agreed with current knowledge, it was also shown that ML models contributed new variables of relevance, which would be interesting to observe in controlled clinical studies (Table 9). For example, pre-eclampsia was found to be predictable based on systemic blood pressure, platelet count, and urinary protein levels as influential variables, with lesser influence found from glucose levels, leukocytes count, serum calcium, and potassium levels (Jhee et al., 2019). Other innovative variables of interest found using ML in the prediction of perinatal complications were newborn sex for the prediction of fetal acidemia (Liu et al., 2019), and father’s nationality and mother’s age for the prediction of provider-initiated spontaneous preterm delivery (Malacova et al., 2020). Nevertheless, some prediction models lack variable measurements, making them impossible to apply in a clinical setting. For example, “weight gain” is mentioned as a predictor for SGA and LGA, but the article does not specify whether it was inadequate or excessive (Kuhle et al., 2018). It is also stated that the underlying disease of the mother influences the delivery initiated by the provider; however, it is not detailed which underlying disease is considered in this association (Khatibi et al., 2019). Also, some studies describe obvious associations, such as low birth weight is associated with SGA, or fetal macrosomia is associated with LGA (Kuhle et al., 2018). pH was also a predictor of fetal acidemia, which is logical since this condition is associated with pH changes (Zhao et al., 2019). Since the engineering team behind these investigations emphasizes these characteristics in the results, without taking this obviousness into account, it is imperative to include clinical experts on women’s health into AI and data science teams.

**TABLE 9:** Main predictive variables for predicting perinatal complications

Prediction	Predictive variables	Machine learning model	Performance	
			AUC	Acc
Premature birth	Gestational diabetes	Set of decision trees, SVM and RF	0.680	81%
	Cardiovascular disease			
	Underlying diseases			
	Maternal age			
	Chronic arterial hypertension			
SGA	Smoking	RF	0.728	79.9%
	A particular values of gestational weight gain	DT	0.718	79.4%
	Low–birth weight newborn	Elastic net	0.748	80.9%
		Gradient increasing machines	0.748	80.5%
		Logistic regression	0.745	81.3%
		Neural network	0.746	81.2%
LGA	Pre-pregnancy BMI	RF	0.745	90.3%
	Gestational weight gain	DT	0.713	80.1%
	Macrosomic newborn in a previous delivery	Elastic net	0.771	91.2%
		Gradient increasing machines	0.766	91.1%
		Logistic regression	0.771	91.2%
		Neural network	0.772	91.4%
Fetal Macrosomia	Greater than 30 years-old	Logistic regression	0.888	ni
	Multiparity	RF	0.990	ni
	A 12 kg total weight gain in pregnancy			
	Abdominal circumference > 95 cm (at last perinatal checkup)			
	Gestation age > 39 weeks			
Pre-eclampsia	At second trimester	Logistic regression	ni	86.2%
	Systolic blood pressure	DT	ni	87.4%
	Serum levels of ureic nitrogen	Naive Bayes	ni	89.9%
	Creatinine in the blood	SVM	ni	89.2%
	Platelet count, serum potassium level	RF	ni	92.3%
	Leukocyte count	Stochastic gradient augmentation method	ni	97.3%
	Blood glucose level			
	Serum calcium and urinary protein levels			
Adverse delivery (preterm, low birth weight, neonatal/infant death, stay in the neonatal intensive care unit) v/s non-adverse delivery	High pre-pregnancy BMI	Logistic regression	ni^a^	ni^a^
		Linear discriminant analysis	ni^a^	ni^a^
	Previous preterm births	Random forest	ni^a^	ni^a^
		Naive Bayes	ni^a^	ni^a^
TOLAC Failure Risk	Parity	Gradient increasing machines	0.793	ni
	Age	RF	0.756	ni
	Vaginal birth with cesarean section in the past Gestational week	RF	0.782	ni
	Minimum gestation week in previous deliveries	AdaBoost set	0.784	ni
	The weight of the newborn from the previous delivery			
	Dilation and head position			
Gestational age (if the newborn will be preterm)	Hypertension during labor	Binary logistic regression model, random forest classification, and generalized additive model	0.868	98.9%
	HIV serological status			
Delivery prediction within 48 h of transfer v/s before 32 weeks gestation	Presence of premature rupture of membranes	Multivariate logistic regression	0.850	ni
	Vaginal bleeding			
	Ultrasound cervical length			
	Gestation week			
	Fetal fibronectin and serum C-reactive protein			
Spontaneous preterm birth	Maternal age	Multivariate logistic regression	0.670	ni
	Black woman			
	Hispanic woman			
	Asian			
	Mother born in the United States			
	Paid delivery by herself or physician			
	Diabetes mellitus			
	Chronic arterial hypertension			
	Thyroid dysfunction			
	Asthma			
	Previous stillbirth			
	Fetal weight loss			
	*In vitro* fertilization			
	Nulliparity			
	Pregnant smoker during the first trimester			
	BMI			
Stillbirth	Current pregnancy complications	Logistic regression	0.834	94.7%
	Congenital anomalies	Decision tree	0.808	99.7%
	Maternal characteristics	Random forest	0.836	99.7%
	Medical history	XGBoost	0.842	99.7%
		Artificial neural networks multilayer perceptron	0.840	99.7%
Prediction of complications in pregnancy: pre-eclampsia, GDM, restriction of fetal growth, macrosomia	Maternal age	Logistic regression	0.770	78.6%
	BMI			
	Newborn weight			
	Results of adverse events in previous pregnancies			
Severe maternal morbidity	Ventilator dependence	Logistic regression	0.937	ni
	Intubation			
	Critical care			
	Acute respiratory failure			
	Ventilation			
	Trauma and postoperative pulmonary failure			
	Fluid and electrolyte disorder			
	Systemic inflammatory response syndrome			
	Acidosis and septicemia			
Fetal acidemia	Maternal age	Deep convolutional neural network	0.978	98.4%
	Gestational age pH			
	Extracellular fluid deficit pC O 2			
	Base excess			
	APGAR 1 min, and 5 min			
	Parity			
	Gestational diabetes			
	Birth weight			
	Child sex			
	Type of delivery			

AUC, area under the curve; Acc., accuracy; SVM, support vector machines; RF, random forest; SGA, small for gestational age; DT, decision tree; LGA, large for gestational age; BMI, body index mass; TOLAC, trial of labor of after cesarean; HIV, human immunodeficiency virus; GDM, gestational diabetes mellitus; ni, not informed.

a This study does not specify either AUC or accuracy. The only performance metric used is sensitivity; logistic regression: 31.9%, linear discriminant analysis: 31.7%, random forest: 30.1%, naive Bayes: 29.2%.

Only 6.4% of the studies were case–control studies, while the vast majority were cohort studies. This may limit the use of these results in clinical practice (Salazar et al., 2019). Only one study was multicenter for predicting neonatal morbidity (Khatibi et al., 2019), representing higher quality evidence. Among the best performing studies, it is noteworthy that most had less than 1,000 patients, and only one based on XGBoost to predict neonatal mortality had over 10,000 patients. This may be risky since the sample size may not be representative for a given geographic group, representing one of the limitations of ML in health (Vayena et al., 2018). Also, another significant limitation of the present systematic review is that all studies included have different baselines, variable inputs, and separate complications (endpoints) assessed in their prediction, making it difficult to compare them.

It is essential to mention that all the studies reviewed have not been applied in a clinical phase; however, the majority mention that to optimize the results obtained, and the models should be used in hospitals or health services that care for pregnant women. Future prospective studies and additional population studies are needed to assess the clinical utility of the model for the real world (Liu et al., 2019; Malacova et al., 2020).

Few systematic reviews have addressed the use of AI in pregnancy. The first one describes how AI has been applied to evaluate maternal health during the entire pregnancy process and helped to understand the effects of pharmacological treatments during this stage (Davidson & Boland, 2020). The second systematic review concluded that using ML algorithms is better than using multivariable logistic regression for prognostic prediction studies in pregnancy care, focusing mainly on decision-making for the medical team (Sufriyana et al., 2020). Furthermore, the third one performed exclusively on neonatal mortality reported that ML models can accurately predict neonatal death (Mangold et al., 2021). Last, the use of modern bioinformatics methods analyzing ML models as non-invasive measures of heart rate variability to monitor newborns and infants was reported (Chiera et al., 2020). Although this body of evidence does not focus on predicting pregnancy complications, it encourages the clinical use of IA to support women’s health during pregnancy.

## Conclusion

In conclusion, the main advantage of interpretable ML applications is that the output is not subjective, due to the fact that it is based on real-world data and results and identifies the most critical variables for clinicians. It is important to continue promoting this field of research in ML in order to obtain solutions with multicenter clinical applicability reduce perinatal complications. AI has the overall potential to revolutionize women’s health care by providing more accurate diagnosis, easing the workload of physicians, lowering health-care costs, and providing benchmark analysis for tests with substantial interpretation differences between specialists. This systematic review contributes significantly to the specialized literature on AI and women’s health.

## Data Availability

The original contributions presented in the study are included in the article/Supplementary Material, further inquiries can be directed to the corresponding author.

## Supplementary Material

Supplementary Figure S1 CASP prediction rule score of each article for bias review.The score for every study included in the systematic review. The maximum score is 22.

Supplementary Table S1 Checklist for compliance with the review based on the PRISMA.

Supplementary Table S2 List of selected items.
