# Supplementary figures and images for: Using Machine Learning to Predict Complications in Pregnancy: A Systematic Review
Source: Front Bioeng Biotechnol. 2022 Jan 19;9:780389. doi: 10.3389/fbioe.2021.780389 (PMC8807522; doi:10.3389/fbioe.2021.780389)

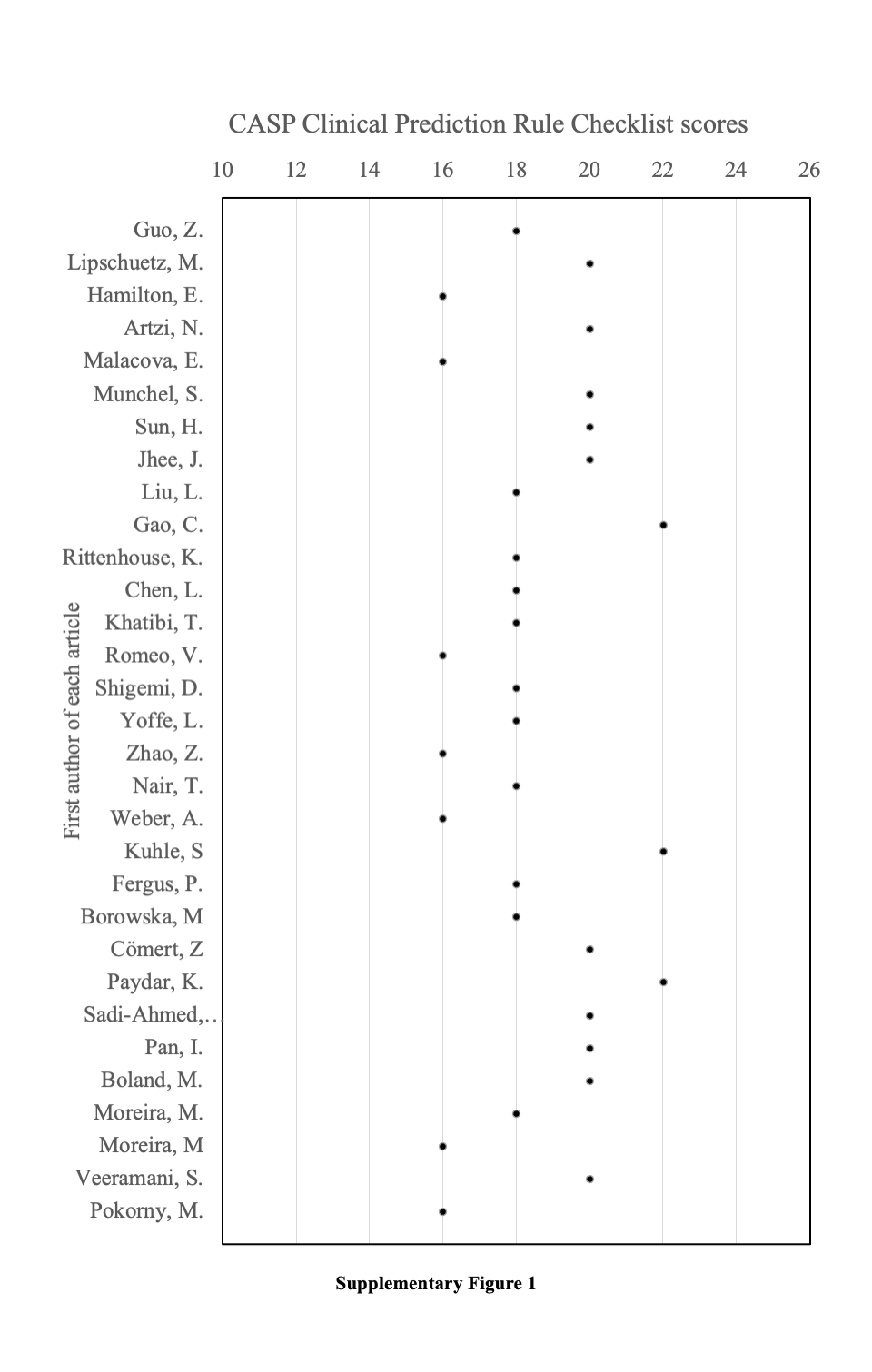

Supplement: Supplementary file 1 [file Image1.tiff]
