# Supplementary material for: Using Machine Learning to Predict Complications in Pregnancy: A Systematic Review
Source: Front Bioeng Biotechnol. 2022 Jan 19;9:780389. doi: 10.3389/fbioe.2021.780389 (PMC8807522; doi:10.3389/fbioe.2021.780389)
Supplement: Supplementary file 2 [file Table1.docx]

***Supplementary Table 1:*** *Checklist for compliance with the review based on the PRISMA method*

| **Section/Topic** | **#** | **Checklist item** | **Reported  on Page #** |
| --- | --- | --- | --- |
| **TITLE** | | | |
| Title | 1 | Identify the report as a systematic review, meta-analysis, or both. | 1 |
| **ABSTRACT** | | | |
| Structured summary | 2 | Provide a structured summary including, as applicable: background;  objectives; data sources; study eligibility criteria, participants, and  interventions; study appraisal and synthesis methods; results;  limitations; conclusions and implications of key findings; systematic  review registration number. | 2 |
| **INTRODUCTION** | | | |
| Rationale | 3 | Describe the rationale for the review in the context of what is already  known. | 3-4 |
| Objectives | 4 | Provide an explicit statement of questions being addressed with  reference to participants, interventions, comparisons, outcomes, and  study design (PICOS). | 4 |
| **METHODS** | | | |
| Protocol and  registration | 5 | Indicate if a review protocol exists, if and where it can be accessed  (e.g., Web address), and, if available, provide registration  information including registration number. | 4 |
| Eligibility criteria | 6 | Specify study characteristics (e.g., PICOS, length of follow-up) and  report characteristics (e.g., years considered, language, publication  status) used as criteria for eligibility, giving rationale. | 4 |
| Information sources | 7 | Describe all information sources (e.g., databases with dates of  coverage, contact with study authors to identify additional studies) in  the search and date last searched. | 4 |
| Search | 8 | Present full electronic search strategy for at least one database,  including any limits used, such that it could be repeated. | 4 |
| Study selection | 9 | State the process for selecting studies (i.e., screening, eligibility,  included in systematic review, and, if applicable, included in the  meta-analysis). | 4 |
| Data collection  Process | 10 | Describe method of data extraction from reports (e.g., piloted forms, independently, in duplicate) and any processes for obtaining and  confirming data from investigators. | 5 |
| Data items | 11 | List and define all variables for which data were sought (e.g., PICOS,  funding sources) and any assumptions and simplifications made. | 5 |
| Risk of bias in  individual studies | 12 | Describe methods used for assessing risk of bias of individual studies  (including specification of whether this was done at the study or  outcome level), and how this information is to be used in any data  synthesis. | 5 |
| Summary measures | 13 | State the principal summary measures (e.g., risk ratio, difference in  means). | N/A |
| Synthesis of results | 14 | Describe the methods of handling data and combining results of  studies, if done, including measures of consistency (e.g., I^2^) for each  meta-analysis. | 5 |
| Risk of bias across  studies | 15 | Specify any assessment of risk of bias that may affect the cumulative  evidence (e.g., publication bias, selective reporting within studies). | N/A |
| Additional analyses | 16 | Describe methods of additional analyses (e.g., sensitivity or subgroup  analyses, meta-regression), if done, indicating which were pre specified. | N/A |
| **RESULTS** | | | |
| Study selection | 17 | Give numbers of studies screened, assessed for eligibility, and  included in the review, with reasons for exclusions at each stage,  ideally with a flow diagram. | 5 |
| Study characteristics | 18 | For each study, present characteristics for which data were extracted  (e.g., study size, PICOS, follow-up period) and provide the citations. | 5 |
| Risk of bias within  studies | 19 | Present data on risk of bias of each study and, if available, any  outcome level assessment (see item 12). | 5-6 |
| Results of individual  studies | 20 | For all outcomes considered (benefits or harms), present, for each  study: (a) simple summary data for each intervention group (b) effect  estimates and confidence intervals, ideally with a forest plot. | 6-SF1 |
| Synthesis of results | 21 | Present results of each meta-analysis done, including confidence  intervals and measures of consistency. | 6-9 |
| Risk of bias across  studies | 22 | Present results of any assessment of risk of bias across studies (see  Item 15). | N/A |
| Additional analysis | 23 | Give results of additional analyses, if done (e.g., sensitivity or  subgroup analyses, meta-regression [see Item 16]). | N/A |
| **DISCUSSION** | | | |
| Summary of evidence | 24 | Summarize the main findings including the strength of  evidence for each main outcome; consider their relevance to  key groups (e.g., healthcare providers, users, and policy  makers). | 9-12 |
| Limitations | 25 | Discuss limitations at study and outcome level (e.g., risk of  bias), and at review-level (e.g., incomplete retrieval of identified  research, reporting bias). | 11-12 |
| Conclusions | 26 | Provide a general interpretation of the results in the context of  other evidence, and implications for future research. | 12 |
| **FUNDING** | | | |
| Summary of evidence | 27 | Describe sources of funding for the systematic review and  other support (e.g., supply of data); role of funders for the  systematic review. | 13 |
