# Supplementary material for: Using Machine Learning to Predict Complications in Pregnancy: A Systematic Review
Source: Front Bioeng Biotechnol. 2022 Jan 19;9:780389. doi: 10.3389/fbioe.2021.780389 (PMC8807522; doi:10.3389/fbioe.2021.780389)
Supplement: Supplementary file 3 [file Table2.docx]

***Supplementary Table 2:*** *List of selected items*

| **First author** | **Article title** | **Year** |
| --- | --- | --- |
| **Guo, Z.** | Whole-Genome Promoter Profiling of Plasma DNA Exhibits Diagnostic Value for Placenta-Origin Pregnancy Complications | 2020 |
| **Lipschuetz, M.** | Prediction of vaginal birth after cesarean deliveries using machine learning. | 2020 |
| **Hamilton, E.** | Estimating risk of severe neonatal morbidity in preterm births under 32 weeks of gestation. | 2020 |
| **Artzi, N.** | Prediction of gestational diabetes based on nationwide electronic health records | 2020 |
| **Malacova E.** | Stillbirth risk prediction using machine learning for a large cohort of births from Western Australia, 1980–2015 | 2020 |
| **Munchel, S.** | Circulating transcripts in maternal blood reflect a molecular signature of early-onset preeclampsia | 2020 |
| **Sun, H.** | Identification of suspicious invasive placentation based on clinical MRI data using textural features and automated machine learning. | 2019 |
| **Jhee, J.** | Prediction model development of late-onset preeclampsia using machine learning-based methods | 2019 |
| **Liu, L.** | An integrative bioinformatics analysis of microarray data for identifying hub genes as diagnostic biomarkers of preeclampsia. | 2019 |
| **Gao, C.** | Learning to identify severe maternal morbidity from electronic health records | 2019 |
| **Rittenhouse, K.** | Improving preterm newborn identification in low-resource settings with machine learning | 2019 |
| **Chen, L.** | Detection of preterm birth in electrohysterogram signals based on wavelet transform and stacked sparse autoencoder. | 2019 |
| **Khatibi, T.** | Analysis of big data for prediction of provider-initiated preterm birth and spontaneous premature deliveries and ranking the predictive features | 2019 |
| **Romeo, V.** | Machine learning analysis of MRI-derived texture features to predict placenta accreta spectrum in patients with placenta previa. | 2019 |
| **Shigemi, D.** | Predictive model for macrosomia using maternal parameters without sonography information | 2019 |
| **Yoffe, L.** | Early diagnosis of gestational diabetes mellitus using circulating microRNAs | 2019 |
| **Zhao, Z.** | DeepFHR: intelligent prediction of fetal Acidemia using fetal heart rate signals based on convolutional neural network. | 2019 |
| **Nair, T.** | Statistical and artificial neural network-based analysis to understand complexity and heterogeneity in preeclampsia. | 2018 |
| **Weber, A.** | Application of machine-learning to predict early spontaneous preterm birth among nulliparous non-Hispanic black and white women | 2018 |
| **Kuhle, S** | Comparison of logistic regression with machine learning methods for the prediction of fetal growth abnormalities: a retrospective cohort study. | 2018 |
| **Fergus, P.** | Machine learning ensemble modelling to classify caesarean section and vaginal delivery types using Cardiotocography traces | 2018 |
| **Borowska, M** | Identification of preterm birth based on RQA analysis of electrohysterograms. | 2018 |
| **Cömert, Z** | Prognostic model based on image-based time-frequency features and genetic algorithm for fetal hypoxia assessment | 2018 |
| **Paydar, K.** | A clinical decision support system for prediction of pregnancy outcome in pregnant women with systemic lupus erythematosus | 2017 |
| **Sadi-Ahmed, N.** | Relevant Features Selection for Automatic Prediction of Preterm Deliveries from Pregnancy ElectroHysterograhic (EHG) records | 2017 |
| **Pan, I.** | Machine learning for social services: A study of prenatal case management in Illinois | 2017 |
| **Boland, M.** | Development of A Machine Learning Algorithm to Classify Drugs Of Unknown Fetal Effect | 2017 |
| **Moreira, M.** | Performance evaluation of predictive classifiers for pregnancy care | 2016 |
| **Moreira, M** | Smart mobile system for pregnancy care using body sensors | 2016 |
| **Veeramani, S.** | Detection of abnormalities in ultrasound lung image using multi-level RVM classification. | 2016 |
| **Pokorny, M.** | Individualized assessment of preterm birth risk using two modified prediction models | 2015 |
